# E-waste recycled materials as efficient catalysts for renewable energy technologies and better environmental sustainability

**DOI:** 10.1007/s10668-023-02925-7

**Published:** 2023-01-18

**Authors:** Rania Seif, Fatma Zakaria Salem, Nageh K. Allam

**Affiliations:** Energy Materials Laboratory, School of Sciences and Engineering, The American University in Cairo, New Cairo, 11835 Egypt

**Keywords:** Sustainability, E-waste, Global market, Recycling, Metal recovery, Hydrometallurgy, Energy conversion, Green hydrogen, Energy storage, Supercapacitor

## Abstract

Waste from electrical and electronic equipment exponentially increased due to the innovation and the ever-increasing demand for electronic products in our life. The quantities of electronic waste (e-waste) produced are expected to reach 44.4 million metric tons over the next five years. Consequently, the global market for electronics recycling is expected to reach $65.8 billion by 2026. However, electronic waste management in developing countries is not appropriately handled, as only 17.4% has been collected and recycled. The inadequate electronic waste treatment causes significant environmental and health issues and a systematic depletion of natural resources in secondary material recycling and extracting valuable materials. Electronic waste contains numerous valuable materials that can be recovered and reused to create renewable energy technologies to overcome the shortage of raw materials and the adverse effects of using non-renewable energy resources. Several approaches were devoted to mitigate the impact of climate change. The cooperate social responsibilities supported integrating informal collection and recycling agencies into a well-structured management program. Moreover, the emission reductions resulting from recycling and proper management systems significantly impact climate change solutions. This emission reduction will create a channel in carbon market mechanisms by trading the CO_2_ emission reductions. This review provides an up-to-date overview and discussion of the different categories of electronic waste, the recycling methods, and the use of high recycled value-added (HAV) materials from various e-waste components in green renewable energy technologies.

## Introduction

The electronics and telecommunications industry has witnessed significant technological and market developments over the past two decades (Khetriwal et al., 2009). Electronics are increasingly used in transportation, healthcare, telecommunications, and security (Nithya et al., 2021). However, these electronic systems become electronic waste (e-waste) when the owner unintentionally disposes them as waste with no intention of reuse. In this regard, e-waste is valuable for achieving a future circular economy and sustainability. Each product has its own material composition that requires recycling strategy. E-waste is an important industry growing at a rate of about 2 million tons (Mt) annually, potentially reaching 74.7 Mt in 2030 (Nithya et al., 2021). This growth is mainly due to economic and rapid technological advances that affect lifestyles and industry. As new devices with the latest features enter the market, consumers will dispose older devices, thus increasing waste rates (Kaliyavaradhan et al., 2022). In terms of quantity, e-waste is one of the fastest-growing global waste streams and significantly affects toxicity (Chung et al., 2011). This massive and increasing amount of electronic waste disposal will have a long-term impact on the planet due to landfill pollution and informal recycling protocols, such as manual sorting, disassembly, and open burning of waste without using safety precautions (Mohammadi et al., 2021). Various electrical and electronic devices (EEEs) contain various toxic substances and precious metals (Gurgul et al., 2018). The heavy metals and toxic substances in e-waste include lead, nickel, antimony, mercury, cobalt, thallium, cadmium, beryllium, polyvinyl chloride (PVC), brominated flame retardants, etc. The e-waste is considered a reservoir of precious and base metals such as silver, gold, copper, and palladium. It serves as a secondary resource for recovery at low operating costs (Kumar et al., 2017a). The valuable recycled metals from electronic waste constitute the essential backbone for a plethora of applications.

Energy is another important factor in our daily life, where fossil fuels are the main source of energy production. The US Energy Information Agency states that fossil fuels accounted for approximately 79% of the total US primary energy production in 2020 (US EPA, 2017). Maintaining today’s fossil fuel consumption rate will deplete petroleum and natural gas resources in the coming 35–70 years (US EPA, 2017). Moreover, greenhouse gases (GHG) and toxic pollutant emissions account for more than 70% of carbon dioxide (CO_2_) emissions (IRENA, 2018). In 2015, the Paris agreement stated the long-term temperature goal of “*holding the increase in the global average temperature to well below 2 °C above pre-industrial levels and pursuing efforts to limit the temperature increase to 1.5 °C above pre-industrial levels, recognizing that this would significantly reduce the risks and impacts of climate change*.”

Consequently, by 2050, the contribution of renewable energy to the global primary supply needs to increase from 12 to 65% to meet the projected energy demand (Abbasi et al., 2020; Gao et al., 2017). The green climate fund, established in 2010, was devoted to helping developing countries to mitigate and comply with climate change and reduce greenhouse gas emissions. Developed countries pledged to allocate US $ 100 billion each year from 2020 to 2025 to support the most exposed and small island developing states in adaptation and mitigation efforts (COP 26 2021). However, the transition to renewable energy in the world is practically limited. For this purpose, the recovered valuable metallic elements from e-waste recycling, such as copper, gold, silver, can be used as excellent catalytic materials in a variety of energy conversion and storage applications, such as oxygen evolution reaction (OER) (Jothi et al., 2018), hydrogen evolution reaction (HER) (Sengeni et al. 2020), methanol oxidation reaction (MOR) (Li & Kanan, 2012), and carbon dioxide reduction reaction (CRR) (Hazra et al., 2019). Specifically, water-splitting (OER and HER) to generate green H_2_ from water is particularly interesting due to its 120 MJ kg^−1^ energy density and CO_2_-free combustion (Karthik et al., 2022).

Corporate Social Responsibility (CSR) restricts firms from continuing the commitment to being socially responsible in contributing to communities and economic growth (Abbas et al., 2019). The environmental analysis emphasizes the efficient management of electronic waste initiated by firms to implement CSR in various schemes for collection and recycling systems. (Al Halbusi et al., 2022; Mubeen et al., 2022). The emergence of social media technology has provided a platform for business firms to interact and communicate with customers, suppliers, retailers, and other stakeholders. Social media marketing application typically facilitates information sharing, and individuals' content generation, where technological innovations have played an essential role in improving firm’s performance (Yu et al., 2022). The efficient collection and recycling management systems will support by the creation of a channel in international carbon market mechanisms. The carbon market can help countries to achieve article 6 of Paris agreement and adopt more ambitious mitigation targets in the waste sector (Deng et al., 2022).

Hence, this review discusses the e-waste market value, e-waste generation, type, and composition. In addition, the available opportunities for e-waste recycling and the categories of recovered value-added materials for energy conversion and storage are highlighted and discussed.

## Global e-waste management market

By 2026, the global electronics recycling market will cost $65.8 billion, with a compound annual growth rate (CAGR) of 12.7%. Metals, one of the segments, are projected to increase at a CAGR of 13.3% and reach $ 53.6 billion by 2026. The electronics recycling market grew to $7.5 billion in 2021. In China, the market size is expected to hit $15.3 billion by 2026, and the annual compound interest growth rate is expected to be 16.3%. Canada and Japan are expected to grow by 9% and 8.9%, respectively. While a CAGR of 12.5% is projected for Germany, other European markets are expected to reach $6.1 billion. Europe is expected to have the largest market share in waste recycling. This is due to the rapid growth and adoption of waste recycling solutions, software, systems, and platforms in a variety of industries, such as security, automotive, marketing, healthcare, retail, information technology, and telecommunications. The USA, Canada, Japan, China, and Europe will promote a 12% CAGR in the global consumer electronics segment. These regional markets have estimated a total of $14.5 billion in 2020 and will grow to $35.4 billion by 2026. China is one of the fastest-growing countries in this regional market cluster. The Asia Pacific market will reach $5.8 billion by 2026, while Latin America will grow at a 16.2% CAGR (Global Electronics Recycling Industry 2022).

## E-waste generation, type, and composition

**Fig. 1:**
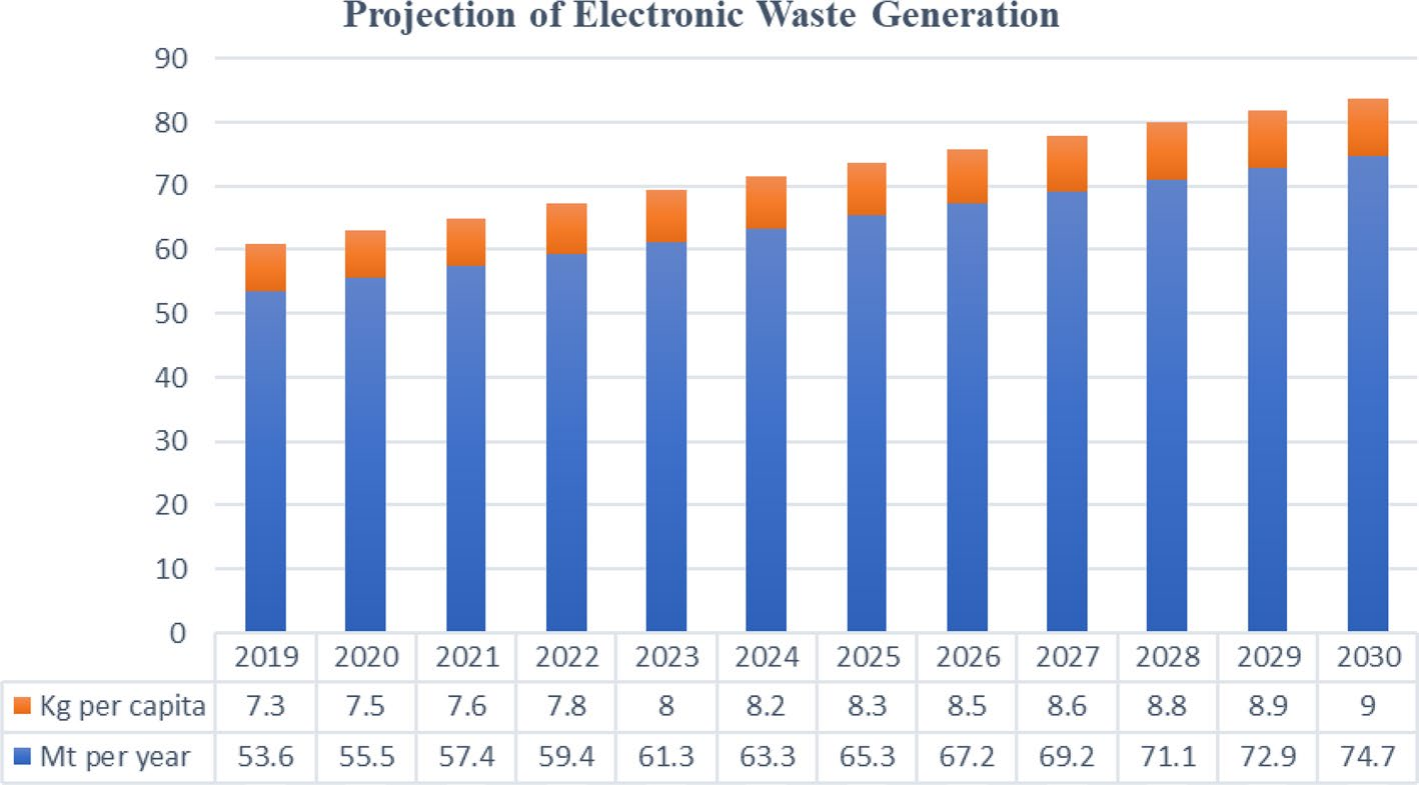
Projection of e-waste generation (Forti et al., 2020)

**Fig. 2:**
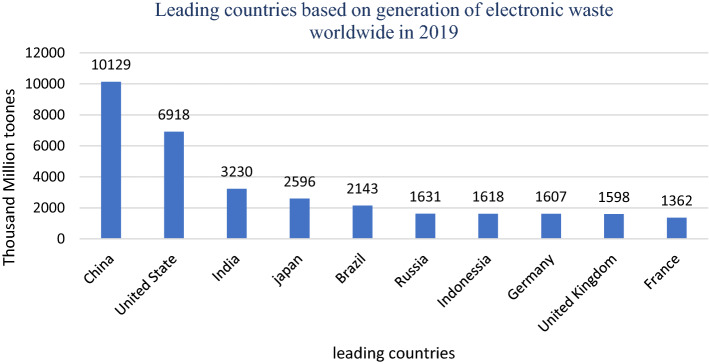
Leading countries in the generation of e-waste worldwide (Forti et al., 2020)

Every year, about 40 million metric tons (Mt) of electronic waste are generated worldwide, accounting for 5% of total solid waste (Kumar et al., 2017a, 2017b). Fig. 1 shows the top countries based on the generation of e-waste around the world (Forti et al., 2020). The European Union’s telephones, televisions, and computers generate about 9 million tons of e-waste. The United Nations Environment Program (UNEP) reported that the electronic waste from mobile phones will increase 18-fold. In contrast, computer e-waste will increase about fivefold, and television e-waste will double in 2020. Fig. 2 shows the annual forecast of e-waste and the future forecast per capita (Forti et al., 2020). In 2019, about 53.6 million tons of electronic and electrical waste were generated worldwide. However, only 17.4% of this waste is adequately recycled (Forti et al., 2020).

a Different categories of electronic waste

**Fig. 3:**
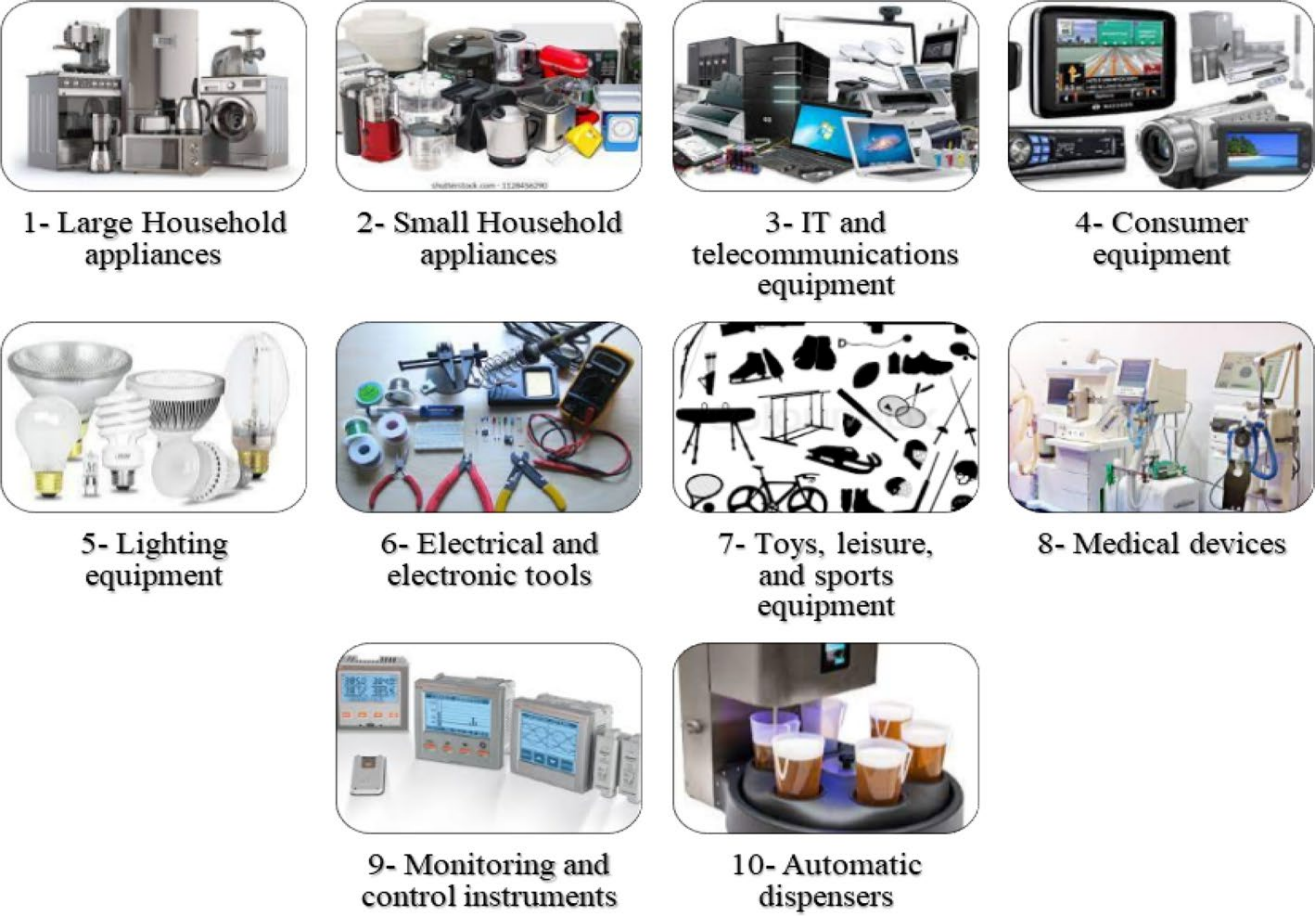
WEEE categories according to the EU directive on WEEE

E-waste includes all non-functional or obsolete electronic items. Based on the European Directives of WEEE 2012/19/EU and 2002/96/EC ((WEEE 2012), E-waste can be classified into ten categories, see Fig. 3. These categories include information technology and communications equipment, large and small home appliances, consumer equipment, non-industrial electrical and electronic appliances, lighting devices, toys, sports equipment, relaxation, non-infected medical devices, control and monitoring units, and automated devices dispensers ((WEEE 2012). About 10% are accessories, 14% are electronic equipment, 34% are communication equipment, and 42% are home appliances (Forti et al., 2020).

b Electronic waste composition

The structural composition of e-waste is generally determined by the electronic device’s manufacturer, its model and type, its date of manufacturing, and its age. IT and communication system chips contain more precious metals than home appliances (Chancerel et al., 2009). A mobile phone, for example, involves more than 40 elements, including precious metals such as gold (Au), silver (Ag), and palladium (Pd), and base metals such as tin (Sn) and copper (Cu) (Liu et al., 2009). Table 1 describes the metal composition of various electrical devices and components (Gautam et al., 2022). Due to the rapid development in electronic industries and high consumer demand, electrical and electronic equipment have a shorter lifetime in developed and developing countries markets, leading to tons of electronic waste. The waste printed circuit boards (PCBs) contain many valuable metals like gold and copper and hazardous materials like lead. Therefore, recycling the metallic and non-metallic fractions from waste PCBs using environmentally friendly and suitable sustainable resource utilization techniques is in high demand. The rapid growth in technology creates significant challenges in developing e-waste management policies. The reuse market, the recycling industry, economic conditions, and waste segregation programs all have an impact on the management system of electronic waste (Gautam et al., 2022).

**Table 1:** Metal content in a plethora of electronic equipment and components (Gautam et al., 2022) with permission from Elsevier

Elements (wt %)	PCB of mobile phones	Smartphones	Desktop personal computer	Laptop	Printer	TV scrap	LCD panel	Internet Routers	DVD player	Portable audio	Hard disk drive	Random access memory (RAM)	Lithium-ion batteries
Cu	37.81	39.5	7	17.61	19.19	3.4	0.0175	21.6333	5	21	5.47	15	1.3
Al	0.61	1.78	–	1.9	7.06	1.2	0.19	5.4433	2	1	54.89	1.4	1
Ni	2.54	1.54	0.8	0.57	5.35	0.038	–	0.4637	0.05	0.03	1.75	4.2	5.5
Zn	1.82	0.67	2.2	0.45	0.73	0.3	0.00171	0.869	–	–	0.67	< 0.01	–
Fe	4.85	0.87	21	3.74	3.56	–	0.133	5.05	62	23	22.48	7	2
Sn	2.55	3.22	1	–	2.03	–	0.00539	3.52	0.2	0.1	0.12	1.9	–
Pb	1.23	0.026	7	2.24	1.01	0.2	–	0.3413	0.3	0.14		0.8	–
As	–	0.0141	14	–	–	–	–	< 0.007	–	–		–	–
Cd	–	< 0.0002	0.01	–	–	–	–	0.0006	–	–		–	–
Cr	–	0.1219	–	0.1	–	–	–	0.0474	–	–		< 0.01	–
Mn	–	–	–	–	–	–	–	–	–	–		0.06	5.8
Hg	–	0.0003	–	–	–	–	–	0.0004	–	–		–	–
Ti	–	0.1	–	–	0.04	–	–	0.1	–	–		–	–
Co	–	–	–	–	0.04	–	–	–	–	–		–	22.7
Li	–	–	–	–	–	–	–	–	–	–		–	3.7
In	–	–	–	–	–	–	0.0178	–	–	–		–	–
Au	0.09	0.1083	0.001	–	0.007	< 0.001	–	0.0199	0.0015	0.0015	0.002	0.08	–
Ag	0.05	0.2773	0.02	–	0.01	0.002	–	0.1213	0.0115	0.0115	0.06	0.04	–
Pd	–	0.00554	–	–	–	–	–	0.00195	0.0004	0.0004	0.002	0.06	–
Pt	–	0.0008	–	–	–	–	–	< 0.0005				–	–
References	(Kasper et al., 2011)	(Holgersson et al., 2018)	(Bhuie et al., 2004)	(Işıldar et al., 2016)	(Yoo et al., 2009)	(Cui & Forssberg, 2007)	(Lahti et al., 2020)	(Holgersson et al., 2018)	(Christian Hagelűken, 2006)	(Christian Hagelűken, 2006)	(Nguyen et al., 2017)	(Landsiedel et al., 2010)	(Atia et al., 2019)

## Electronic waste management system

**Fig. 4:**
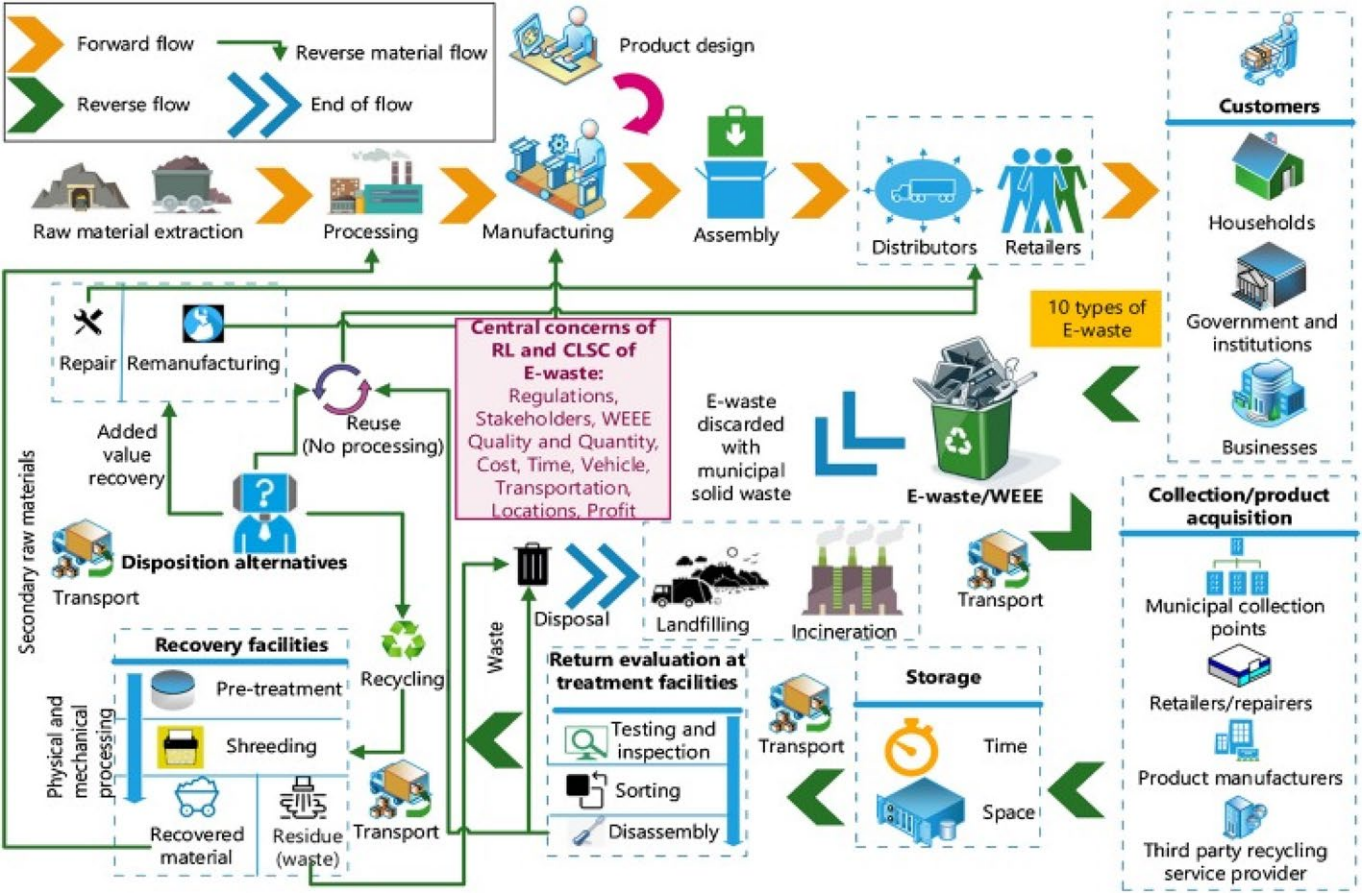
Closed Loop supply chain of e-waste (Islam & Huda, 2018) with permission from Elsevier

Generally, a waste management system consists of three essential steps: collection, pretreatment (dismantling, sorting, and size reduction), and recovery, as seen in Fig. 4 (Islam & Huda, 2018). Recycling e-waste can be informal or formal to extract valuable metals. Informal recycling refers to open burning to recover economically valuable metals, such as copper, aluminum, gold, lead, mercury, cadmium, palladium, and platinum. In addition, several chemicals, such as cyanide, HNO_3_, HCl and H_2_SO_4_, are highly toxic when used informally.

### Proposed system to enhance the collection system worldwide

**Fig. 5:**
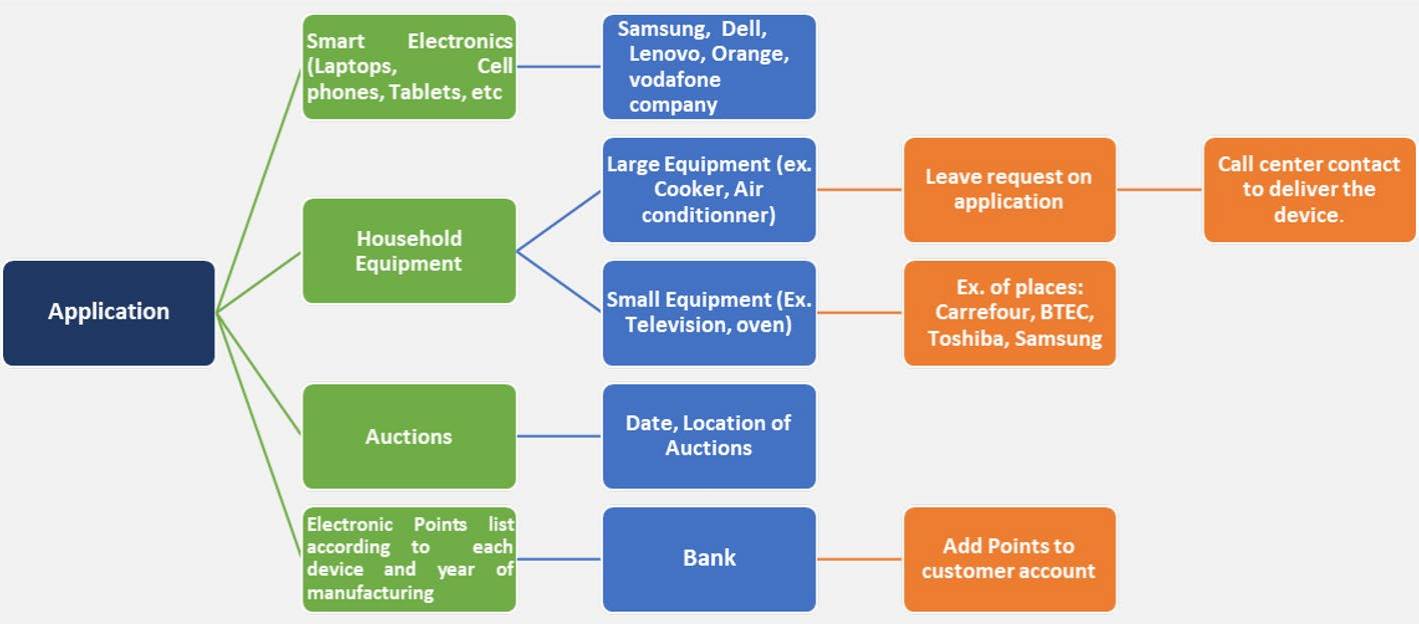
Design application for the collection of e-waste

Improving e-waste management begins with establishing a proper collection system. Everyone can easily download mobile phone applications from the Google play store. Creating an application allows all stakeholders who benefit from it to collect more e-waste. Figure 5 depicts a description of a design application for e-waste collection. The application is classified into four categories based on the type of e-waste and the beneficiary customer. The first category includes smart electronics (laptops, cell phones, tablets, etc.) as well as places to sell old electronics. The second category is divided into two subcategories: large-size equipment and small-size equipment. The customer can submit a request on the application for large-size equipment, and the call center will respond for more information on how to receive the old equipment. The small-size equipment can easily be dropped off by the customer to the available locations. The third category is more specialized in collection agencies, private sectors, and governmental agencies. This category contains the locations and dates of auctions that assist recycling factories in collecting raw materials. The final category includes a list of electronic points for each device. This category consists of the points equivalent for each device to sell and the year in which the device was manufactured. The customer can only replace the device with points for locations agreed upon and found via the application. The points can be exchanged for money by adding them to the customer's card, used in any purchasing market.

### Recycling processes of different types of e-waste

In Egypt, Twelve WEEE management firms have obtained environmental approval from the Egyptian Environmental Affairs Agency (EEAA) and an operational permit from Industrial Development Authority and are listed as a formal sector. Ten are based in the Cairo governorate, and two are located in the Alexandria governorate. Four WEEE management firms conduct in-depth dismantling, and two show complete recycling on the WEEE, in which mechanical and chemical processes are involved in the recycling process. The in-depth dismantling WEEE management firms mainly collect, dismantle, and sort extracted fractions for resalable in the local market. In contrast, other bits are exported abroad to China, Korea, the USA, Belgium, and Italy. Specialized WEEE refining carries forward shredding, smelting and refining mechanical and chemical processes on exported WEEE fractions (United Nations Development Programme 2015).

Egypt’s recycling and recovery are limited due to the lack of environmental policies integrated with specific laws governing the disposal and management of WEEE, the lack of precise databases and accurate mapping of the e-waste in Egypt, illegal incineration and dumping of hazardous e-waste with other non-hazardous/hazardous solid waste, releasing highly toxic emissions of furans, dioxins, and other gases that possess imminence risks on the environment and human health. Furthermore, the lack of technical expertise and equipment related to the recycling of e-waste (Abdelbasir et al., 2018). Currently, recycling is more expensive than exporting e-waste or metal concentrate due to the lack of local integrated treatment facilities and economic factors. Only 15–20% of Egypt’s electronic waste is locally recycled, according to the United Nations Environment Program (UNEP). The rest will go directly into incinerators and landfills (United Nations Development Programme 2015). Improving the electronic waste management system in Egypt requires raising public awareness among the relevant stakeholders, establishing an e-waste database, formulating national strategies and legislation, and gaining technical expertise in various recycling. Furthermore, capacity building with the best environmental practices (BEP) and technology available (BAT) (Abdelbasir et al., 2018).

Recycling of e-waste involves three significant steps (Atia et al., 2019): (a) Dismantling: the main process in e-waste recycling that focuses on identifying valuable or hazardous components for special treatment, (b) Upgrading: using metallurgical/mechanical processing protocols to prepare the desirable materials for a refining process, and (c) Refining: in the final step, purifying/retreating the recovered materials using chemical/metallurgical processing protocols to be functional for their original use. Disassembly and mechanical processes are primarily employed to pre-treat the e-waste to improve the content of the desirable materials. Several researchers have thoroughly reviewed and investigated the mechanical recycling of electronic waste (Cui & Forssberg, 2003; Matsukami et al., 2015). On the other hand, mechanical recycling is inefficient at recovering precious metals. Finally, pyrometallurgical and hydrometallurgical processes are used to dissolve or melt the recovered metals to remove any existing impurities. This step involves a number of chemical reactions (Cui & Forssberg, 2003). This review provides a comprehensive literature survey on the methods used to recycle lithium-ion batteries, precious metals from mobile phones, and copper recovery from computer-printed circuit boards to provide a detailed research status for future studies.

#### Hydrometallurgical recycling of precious metals from mobile phones PCBs

The number of mobile phone subscriptions globally has exceeded the total population. In 2017, mobile phone subscriptions for the global population reached 1.04 per capita (Tan et al., 2018). Due to technological developments, social–psychological reasons, and upgrades, the lifespan of a mobile phone may be less than two years (Wilson et al., 2017). In 2017 alone, 800 million mobile phones were discarded in China (Guo & Yan, 2017). The massive consumption and rapid obsolescence of mobile phones pose serious challenges to the management of used mobile phones (WMP). WMPs are rich in resources, and their metal content is much higher than natural ores (Zeng et al., 2016). On average, each ton of WMP contained 0.15 kg of palladium, 0.347 kg of gold, 1 kg of antimony, 3.63 kg of silver, 6 kg of lead, 10 kg of tin, 15 kg of nickel, and 128 kg of copper (Valero Navazo et al., 2014). Recycling elements from electrical and electronic waste equipment is more cost-effective than mining (He et al., 2018).

High-quality precious metals, for example, silver and gold, are extracted via hydrometallurgical processing. The procedure is usually performed using different reagents. Cyanide leaching was the main reagent used for precious metals. The reaction is selective and leads to stable dicyanoaurate or dicyanoargentate complexes (Wang et al., 2021). Due to its toxic nature, cyanide has recently been replaced by aqua regia, thiosulfate, and thiourea. Table 2 shows the different leaching agents used to recover precious metals from waste mobile phones (Ding et al., 2019).

**Table 2:** Different processes for recovering precious metals (Ding et al., 2019) with permission from Elsevier

Leaching agent	Process conditions	Metals recovered	References
DMF-CuCl_2_–CaCl_2_ (DMF: dimethylformamide) system in mild aqua regia	Temp. 75 ℃, leaching time 120 min., L/S ratio 500, cucl_2_ content 0.75 mol/L	Au 99%	(Wang et al., 2021)
Na_2_S_2_O_8_-KI leaching process combined with H_2_SO_4_–H_2_O_2_	0.02 M Na_2_S_2_O_8_, 0.15 M KI, 40 ℃, a solid–liquid ratio of 1: 20 (g/mL), and stirring speed = 200 rpm, 120 min leaching time	Au	(Pan et al., 2022)
7 g e-waste, 0.2 mol SC(NH_2_)_2_, 0.5 mol HCl, 0.1 mol FeCl_3_	Temp 25 ℃, Time 2 h, L/S ratio 40	Au 54.81%, Ag 20.72%, Pd 29.6%	(Diaz et al., 2016)
Ag iodine/iodide 1:6	Temp 25 ℃, Time 1 h and 30 min, S/L ratio 1/8	Ag 99%	(Xiu et al., 2015)
Pd, Au iodine/iodide 1:5; pH = 9,	Temp. 25 ℃, Time 2 h, S/L ratio 1/10	Pd 97.2%, Au 98.5%	(Xiu et al., 2015)
100 g/L H_2_SO_4_, 75 g/L NaCl, 25 g/L NaClO_3_	Temp 40 ℃, Time 2 h, S/L ratio 1:8	Au 99.6%	(He & Xu, 2015)
72.71 mmol Thiosulfate, 10.0 mmol Cu^2+^, 0.266 mol NH_3_	Temp 40 ℃, Time 5 min	Au 91%	(Ha et al., 2014)
Copper was removed before the experiment. Iodine 3%, H_2_O_2_ 1%, pH = 7	Temp 35 ℃, Time 4 S/L 3:20	Au 99.98%	(Sahin et al., 2015)
0.13 M ammonium thiosulfate, 0.02 M Cu^2+^	Temp 20 ℃, S/L 50, ratio (g L^−1^) 50, pH = 10, Stirring (rpm) 180, Duration (min) 120	Au (70.5%)	(Camelino et al., 2015)
0.12 M thiosulfate, 0.2 M ammonium, 20 mM Cu^2+^	Temp 30 ℃, S/L, ratio (g L^−1^) 25, pH = 10, Stirring (rpm) 180, Duration (min) 240	Au (75%)	(Kasper et al., 2018)
0.65 M thiourea, 1.5 M Fe^3+^, H_2_O_2_	Temp 70 ℃, S/L, ratio (g L^−1^) 200, pH = 1	Au (95%), Ag (93%) Pd (99%)	(Quinet et al., 2005)
0.5 mol/L HNO_3_, potential 0.35 V, electrodeposition	Time 5 h, current efficiency 63.48%	Ag 97.72%, Pd 98.05%	(Liu et al., 2022)
Sulfuric acid; thiourea	(1) copper removing: 5% H_2_O_2_; 2 M H_2_SO_4_; 25 and 30 ℃, 200 rpm for 3 h (2) gold leaching: 20 g/L thiourea; 1/10 S/L ratio; 0.5 M H_2_SO_4_; and 6 g/L ferric ion, pH = 1; 500 rpm; room temperature	79.1 mg/L Au, 121.1 mg/L Ag	(Birloaga et al., 2014)
Thiourea – ferric sulfate leaching	Leaching in thiourea with ferric sulfate, followed by filtration	Au (69.36%), Cu (100%), Ag (100%)	(Lee et al., 2011)
HNO_3_ & Aqua Regia	Dissolving the powder in HNO_3_ then aqua regia, followed by precipitation in hydrazine and oxalic acid	Au 99.9%	(Sahan, 2016)

#### Recovery of copper from PCBs

Copper is the main component used in printed circuit boards. It accounts for about 25–30% of the total metals used, which is much higher than copper ore, representing 20–40 fold (Xiu et al., 2017). Several techniques for recovering and reusing copper from PCB waste have been proposed (Xiu et al., 2017). Pyrometallurgy (Flandinet et al., 2012), hydrometallurgy (Fogarasi et al., 2015), and mechanical methods (Guo et al., 2011) are among the techniques proposed. Among these technologies, hydrometallurgical processes mainly focused on the leaching of copper with high metal selectivity, no dust/gas formation, relatively low capital costs, functional selectivity, and suitability for small-scale applications are alternatives for the treatment of PCBs waste (Birloaga et al., 2013). In previous reports, different lixiviant systems, including hydrochloric acid, sulfuric acid, nitric acid, ammonia, iodide, cyanide, thiosulphate, thiourea, and microbes, were investigated.

Metallic nanoparticles have unique structures, large surface area, high thermal and mechanical stability, and unusual chemical, optical, magnetic, and catalytic properties, making them the focus of many investigations. Cu NPs have been used in electronic, pharmaceutical, cosmetic energy, catalytic (Wang et al., 2016), solar energy conversion (Sahai et al., 2016), material additives (Jiao et al., 2016), negative electrode material of lithium-ion batteries (Poizot et al., 2000), and microelectronics (Li & Chen, 2014). Several research groups have recently proposed different methods for recover ultrafine copper materials. It is a promising strategy for increasing the added value of copper products derived from waste PCBs. Chemical reduction (Yang et al., 2012), electrochemical process (Chu et al., 2015), and electrokinetic process (Xiu & Zhang, 2009) are among the methods proposed.

Surfactants, reductants, and stabilizers must be used in the proposed processes, among other chemical reagents. Conductive pastes made of copper nanopowder have been used to form thick-film conducting electrodes or patterns in PCBs and multilayered electronic parts (Hai et al., 2007). A current trend in the electronic industry is the replacement of precious metals, such as Ag, Au, or Pd, with Cu nanopowder to produce conductive paste for hybrid integrated circuits and the metallization of multilayer ceramic capacitors (MLCC) (Hai et al., 2007). For example, Cu nanopowder is a potential raw material for the future additive manufacturing industry.

Nowadays, electrospinning is an economical and industrially scalable technique for producing nanofibers (Khalil et al., 2014; Mohamed et al., 2017). Electrospinning was used to create Cu-NPs using a precursor composed of copper acetate and polyvinyl alcohol at 20–29 kV (Khalil et al., 2014). The results showed that Cu NPs had an average diameter between 30 and 70 nm, while demonstrating a good yield and size distribution. Table 3 shows different hydrometallurgical processes for copper recovery from electronic waste.

**Table 3:** Different processes for the recovery of copper from E-waste

Leaching agent	Process conditions	Metals recovered	References
50 ml con. HCl (37%), 50 ml con. HNO_3_ (63%),	30 ml of 3 M NaOH added dropwise, black precipitate drying for 6 h at 80 °C, then calcination for 4 h at 400 ℃	CuO Nanoparticles	(Rajkumar et al., 2022)
0.2 M H_2_O_2_ and 2 M H_2_SO_4_	S/L 1/100, time 150 min, temp at 25, 30, 35, and 45 ℃	Cu	(Dávila-Pulido et al., 2021)
H_2_SO_4_ leach solution	Extractant LIX 84 IC, Con. 10%, Diluent Kerosene, pH 2.5	Cu 99.99%	(Kumari et al., 2016)
Ammonia-ammonium leach Solution	Extractant LIX 84, Con. 50%, Diluent Kerosene, pH 10	Cu 99.6%	(Yang et al., 2012)
Ammoniacal ammonium salts leaching	Temp 25 ℃, S/L 1/10, ascorbic acid as a reducing agent, CTAP	Cu 99.99%	(Seif El-Nasr et al., 2020)
Digested metal powders of WPCBs by microwave aided HNO_3_—H_2_O_2_—HF system	Slurry electrolysis	Cu 99.3%	(Zhang et al., 2017)
H_2_SO_4_, H_2_SO_4_ + HCl, HCl, HCl + HNO_3_	HNO_3_ + HCl leaches Cu with high percentage while H_2_SO_4_ showed the lowest leaching after 120 min	Cu 92.7%	(Vijayaram R et al. 2013)
Copper foil liberation by chemical-ultrasonic treatment and dissolution in H_2_SO_4_ acid	Ascorbic acid reduction in the presence of cyclodextrin	Cu 90%	(Tatariants et al., 2018)
Using six ionic liquids to leach Cu from WPCBs	Leaching experiments were carried out in 250-mL glass conical flasks. Parameters examined: WPCBs particle size, H_2_O_2_ amount, acid concentration, and time at 40–70 ^0^C (250 rpm)	Cu 99.99%	(Chen et al., 2015)
(i) H_2_SO_4_; (ii) H_2_SO_4_ + H_2_O_2_	Temp. (75 °C), time (4 h) and solid/liquid rate (1:10)	Cu 98.46%	(Silvas et al., 2015)
H_2_SO_4_	70 °C, S /L = 1:20, air flux = 3 L/min, 400 rpm, 4.5 h	Cu 95.72%	(Guo et al., 2020)
Mechanical treatment, 3 mol/L ammonia water, 2 mol/L ammonium chloride were used as leaching agents	Temp 45 °C, and the leaching time was set at 20, 40, 60, 80, 100, 120, 140, 160, 180, 200 min. 10 mL of leaching solution was used, CTAB, glucose was used for the pre-reduction process at 85 °C. Ascorbic acid reducing agent, and copper was placed in a vacuum drying oven for 24 h at 60 °C to avoid oxidation	Cu 99.9%	(Zhu et al., 2021)
Potassium persulfate (K_2_S_2_O_8_)	Variation in Temp. leaching 25, 35, 45, and 55 °C, S/L 1/100, constant magnetic stirring (300 rpm), Cooling recrystallization	Cu 99.9%	(Liu et al., 2019)

#### Recycling of lithium-ion batteries (LIB).

**Fig. 6:**
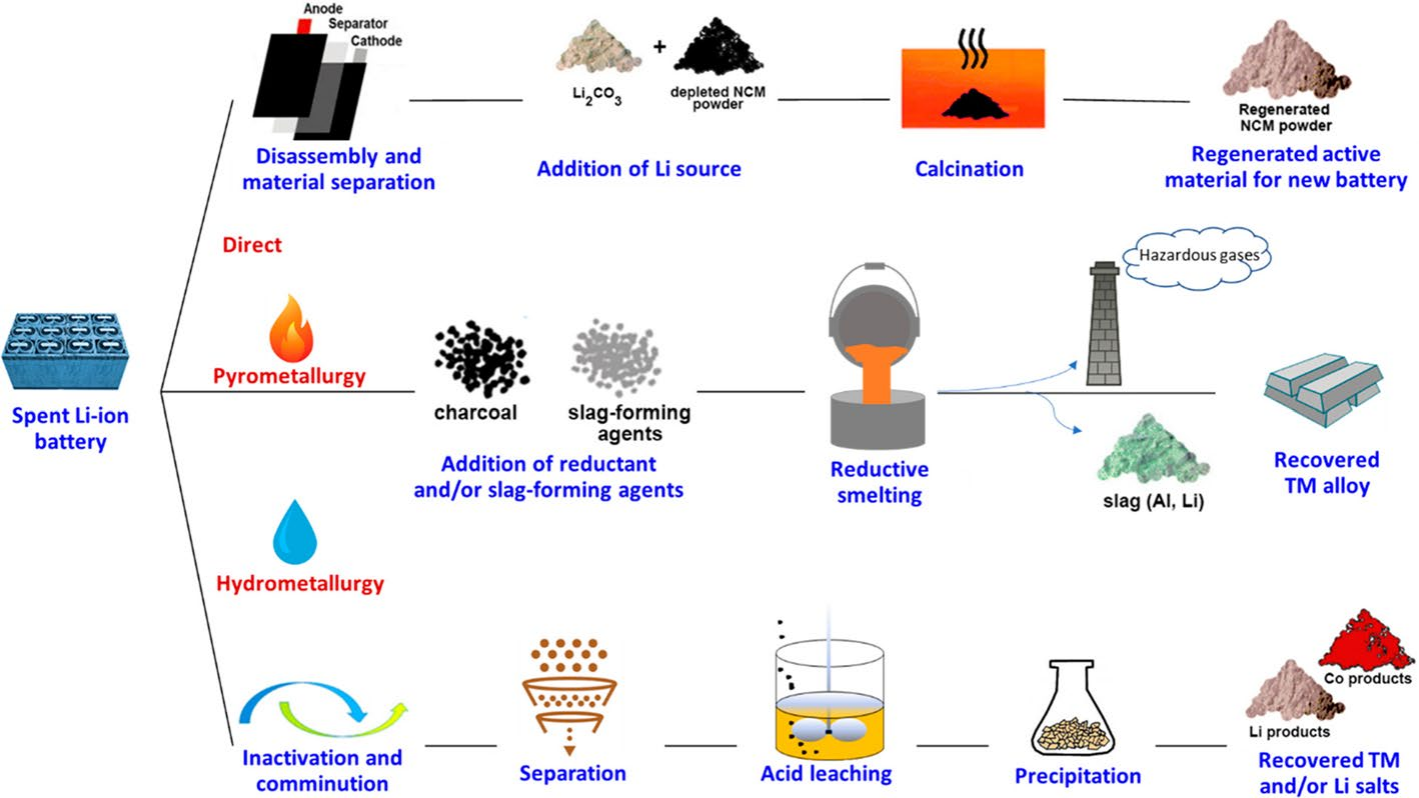
Typical direct, pyrometallurgical, and hydrometallurgical recycling methods for recovering Li–ion battery active materials (Baum et al. 2022). With permission from the American Chemical Society

The global lithium-ion battery (LIB) market is projected to grow from $ 41.1 billion in 2021 to $ 116.6 billion in 2030, with a 12.3% CAGR (Zheng et al., 2018). As a result, the global mining production of cobalt for LIBs fabrication has exceeded 140,000 metric tons per year [98]. Furthermore, cobalt is produced as a byproduct of copper and nickel mining, accounting for 98 % of total cobalt production. However, the current rise in demand for electric vehicles (EVs) has resulted in an extreme shortage of cobalt supply, resulting in a 311% price increase from $12.01/lb (2016) to $37.43/lb (2018) (Raj et al., 2022). Industrially, various Lithium-ion-based metal oxides, such as Li Ni_1–x–y_Co_x_Mn_y_O_2_ (NCM), are rapidly being used in electric vehicles to produce high-energy electrode material (> 600 Wh kg^–1^) (Pender et al., 2020). LIBs must go through several processes before they can be reused or recycled due to their various compositions and complex structure. The LIBs must first be classified before they can be discharged or deactivated, disassembled, and separated. Then, as shown in Fig. 6 (Baum et al. 2022), LIBs can be recycled using pyrometallurgy, hydrometallurgy, or a combination of methods. The direct process of removing cathode material for reuse or reprocessing requires disassembling the LIB to obtain usable battery material (Baum et al. 2022). Heating is used in pyrometallurgy to convert the metal oxides used in battery materials into their metal compounds or metal counterparts (Zhou et al., 2021). After pretreatment, the battery materials are heated in a vacuum or inert atmosphere to prepare mixed metal alloys containing Co, Ni, Cu, Fe, and slag containing Li and Al, depending on the battery composition. Pyrometallurgical methods require simpler pretreatment protocols, such as shredding or crushing, to prepare batteries for recycling and require fewer different processes to recycle LIB of various compositions, shapes, and sizes.

Hydrometallurgical methods mainly use aqueous solutions to extract and separate metals from LIBs. The pretreated battery materials with previously removed Al and Cu current collectors are typically extracted with H_2_SO_4_ and H_2_O_2_. HCl, HNO_3_, and oxalic or citric acids. Metals are extracted into a solution and selectively precipitated as salts via pH control or extracted using organic solvents containing extractants such as dialkyl phosphates or phosphinate (Baum et al. 2022).

A LIB is typically made of an anode, a separator, a cathode, an electrolyte, an outer case, and sealing parts. Spent LIBs typically contain 5–20% cobalt (Co), 5–10% nickel (Ni), and 5–7% lithium (Li), which are higher than those found in natural ores, 5–10% other metals such as copper, aluminum, and iron. In addition, 15% organic compounds, 7% plastic, with slight variation depending on the manufacturer (Zheng et al., 2018). The recovered metals from spent LIBs, such as Li, Ni, Co, and Mn, have a significant economic impact (Zheng et al., 2018). LIB cathode materials are mostly Li intercalation oxides, such as LiMn_2_O_4_, LiNiO_2_, LiFePO_4_, LiCoO_2_, and Li Ni_x_ Co_y_ Mn _1-x-y_ O_2_ (Wang & Sun, 2015). Also, Li Ni_x_ Co_y_ Mn_1-x-y_O_2_ has emerged as the most prominent LIB cathode material. This is due to its large discharge capacity, low cost, stable structure, and good cyclic performance (Wang & Sun, 2015). On the other hand, the anode (negative active material) contains graphite as an active material with a copper foil coating; an electrolyte is required for ion transfer. It acts as a medium through which ions are moved from one electrode to the other, converting chemical energy into electrical energy (J. Baum et al. 2022). LiPF_6_, LiBF_4_, LiCF_3_SO_3_, or Li (SO_2_CF_3_)_2_ are the electrolytes used in LIBs (Ren YH, Wu BR, Yang CW, Wu F). Many studies have discussed the leaching of spent LIBs using inorganic acids, electrochemical leaching, organic leaching, and ammonia leaching. Table 4 lists some of these reports and the corresponding experimental conditions for recovering materials from Lithium-ion batteries.

**Table 4:** Various Methods for leaching Lithium-ion batteries (Yang et al., 2021) with permission from Elsevier

Recovered Materials	Reducing and leaching agents	Conditions	Recovery efficiency	References
*1 Electrochemical Leaching*				
Li Ni Mn Co Hydroxide	3 M HCl and H_2_O_2_ 35% agitation	80 °C, 2 h, pH 6–7, electrochemically deposited onto nickel foam	100% recovery of complex composed of Li Ni Mn Co Hydroxide has great efficiency in Supercapacitor	(Mesbah et al., 2020)
LiCoO_2_	1.25 M malic acid	70 °C, 3 h, and pulp density of 8 V, 0.1 g	Co 90%, Li 94%	(Meng et al., 2018)
*2 Inorganic acid leaching*				
Li, Co	1.0 M maleic acid and 0.3 M SnCl_2,_ H_2_O_2_	20 g·L^−1^, 60 °C, 40 min	Li 98.67%, Co 97.5%	(Sun et al., 2021)
Li, Co	1.0 M HNO_3_	75 °C, S/L ratio 1/50, 1 h, and reducing agent 1.7% (v/v) H_2_O_2_	85% Li, Co	(Lee & Rhee, 2002)
LiCoO_2_	5 vol% H_2_O_2_ + 2 M H_2_ SO_4_ as reducing agent	75° C, 1 h, 100 g•L^−1^	Co 99.1%, Li 70%	(Jha et al., 2013)
	0.02 mol/L glucose + 1.5 M H_3_PO_4_ as a reducing agent	80 °C, 2 h, pulp density 2 g•L^−1^	Co 98%, Li ∼100%	(Meng et al., 2017)
Li Ni_x_Co_y_Mn_z_O_2_	1 vol% H_2_O_2_ + 1 M H_2_ SO_4_	40° C, 1 h, 40 g•L^−1^	Li, Ni, Co, Mn ∼ 100%	(He et al., 2017)
	4 M HCl	25° C, 120 gL^−1^	81%Co, 98.0% Li, 75.5%Mn, 98% Ni	(Xuan et al., 2019)
	0.4 M Citric acid + 0.2 M H_3_ PO_4_	90 °C, 30 min, pulp density 20 g•L^−1^	91.63% Co, 100% Li, 93.38% Ni, 92% Mn	(Zhuang et al., 2019)
LiFePO_4_	H_2_O_2_ + 0.3 M H_2_SO_4_ (H_2_O_2_/Li molar (2.07)	2 h, 60 °C	96.85% Li, 1.95%P, 0.027% Fe,	(Li et al., 2017)
Li (NiCoAl)O_2_	4 M HCl	90 °C, 50 g•L^−1^, 18 h	Li, Co, Ni, Al ∼ 100%	(Joulié et al., 2014)
Li, Co	5% NaOH 4 mol/L H_2_SO_4_,1.0% H_2_O_2_	85 °C, 10 g L^−1^; 4	95% Co, 96% Li	(Xiao et al., 2017)
*3 Organic acid leaching*				
LiCoO_2_	4 vol% H_2_O_2_ + 1.5 M succinic acid	70 °C, 15 g•L^−1^, 0.67 h	Li > 96, Co ∼100	(Li et al., 2015)
	0.75 M 3 vol% H_2_O_2_ + Benzenesulfonic acid	90 °C, 15 g•L^−1^, 80 min	Li 99.58%, Co 96.53%	(Fu et al., 2019b)
	6 vol% H_2_O_2_ + 2 M DL-malic acid	95 °C, 20 g•L^−1^, 60 min	Li 93.22%, Co 90.57% Mn 99.53%	(de Oliveira Demarco et al. 2019)
	1.5 M formic acid + 1.3 M Benzenesulfonic acid	50 °C, 30 g•L^−1^, 40 min	Li 99%, Co 97%	(Fu et al., 2019a)
	0.02 M ascorbic acid + 0.5 M glycine	80 °C, 2 g•L^−1^, 6 h	Co > 95	(Nayaka et al., 2016)
LiNi_x_Co_y_ Mn_1-x–y_ O_2_	0.5 M Citric acid	90 °C, 80 min, 80 g•L^−1^	91% Li, 94% Ni, 89% Mn, 90% Co	(Meng et al., 2020)
	1 M Acetic acid + 3 mL H_2_ O_2_	70 °C, 20 g•L^−1^, 60 min	98.83% Li, 97.93 Ni, 97.85% Co, 97.74% Mn	(Li et al., 2018)
Mn, Li, Ni	10 ml/L^−1^ formic acid + 0.2 mol L^−1^) H_2_O_2_	80 °C, S/L ratio 50 g L^−1^, 3 h	99% Li 98% Ni 99%Mn	(Zeba et al., 2022)
*4 Ammonia leaching*				
Li(Ni_1/3_Co_1/3_Mn_1/3_)O_2_	1.5 M (NH_4_)_2_ SO_4_, 4 M NH_3_, 0.5 M Na_2_ SO_3_	80° C, 10 g•L^−1^, 5 h	Mn 1.36%; Co, Ni, Li > 98%; Al 0.02%	(Zheng et al., 2017)
Li, Ni, Co and Mn	(NH_4_)_2_SO_4_, NHS), sucrose, H_2_SO_4_	30 min, pH = 2.0, 25° C, S/L = 25 g/L	Li 97.1% Ni 94.0% Co 87.6% Mn 93.8%	(Liang et al., 2022)
Co, Ni, Mn	1.5 M ammonium citrate, 3 M ethylenediamine	2 h, 30° C, 50:1 L/S	Ni 99.5% Co 99.4% Mn 99.9%	(Su et al., 2022)

## Application of materials extracted from electronic waste in green technologies

One main challenge that currently faces scientists is the sustainable e-waste management. The annual global amount of generated e-waste is expected to increase by 2030, reaching 74.4 by 2030 (Forti et al., 2020). Landfilling, a technique used to get rid of the e-waste, generates vast amounts of methane that contributed to the global warming problem over 100 years by 21 times more than that of CO_2_ (Pariatamby et al., 2015). Landfilling is responsible for almost 14% of global methane greenhouse gas emissions (Das et al., 2019). Due to the rigorous environmental regulations, the traditional approaches used for e-waste disposals, such as exporting abroad, burning using incinerators or landfilling disposal, are no longer valid options (Das et al., 2019).

Accordingly, the precious elements in the e-waste and the rising demand for the materials, along with the complications of the presently attainable raw materials, make the extraction of the valuable metals recycled from the e-waste an attractive alternative both economically and environmentally in diverse energy conversion and storage technologies. The metals extracted from the e-waste can be used as a water-splitting catalyst to generate hydrogen under specific conditions. Sustainable recycling of e-waste addresses the shortage of raw materials and minimizes energy consumption during the production of the raw materials while handling environmental concerns associated with hazardous materials generated from electronic waste streams (Das et al., 2019). Energy consumption predominantly contributes to greenhouse gas emissions since the recorded CO_2_ emissions from fossil fuel combustion in 2018 was 33.1 Gt in 2018 (Zedalis, 2017). Therefore, replacing fossil fuels with an alternative clean energy source will be vital in lessening global CO_2_ emissions. The following section of the review elaborates on the recovered metals from e-waste and their application in energy conversion and storage technologies.

- a Hydrogen production

Waste-to-Hydrogen is the process that emphasizes producing hydrogen from recycling waste materials. Recently, there has been high interest from both governments and industry sides for utilizing hydrogen as a replacement for fossil fuels due to its unique properties (Lui et al., 2020). Hydrogen produces zero greenhouse gas emissions when combusted since it produces only water in the vapor phase, which could significantly enhance the main energy sector decarbonization problem. Nevertheless, the currently used methods for hydrogen production are around 96% depend on fossil fuels, while almost 0.04% comes from renewable energy sources. In addition, 96% of hydrogen obtained from natural gas, petroleum oil, and coal leads to an annual production of CO_2_ emissions of 560 million tons, representing 1.7% of the global CO_2_ emissions (Zedalis, 2017). Thus, renewable energy, such as wind and solar energy, is used to attain the advantages of using hydrogen as a clean, efficient, and versatile energy source. Hydrogen production from electrochemical water splitting is an example of producing hydrogen from a green energy source. This method includes running electricity through water using an appropriate catalyst to produce hydrogen and oxygen.

### Green technologies for green hydrogen production

Water electrolysis is an efficient, promising, and simple technique for the production of pure hydrogen (Anantharaj et al., 2018). Nonetheless, external power is required to recognize the water-splitting process, rendering the water-splitting process economically inefficient. Instead, capturing and storing renewable energy from the surrounding environment, for instance, wind, solar, tidal or thermal energy, can be used for the electrolysis that could eliminate or reduce the need to utilize the external power supply (Wang and Li and Kazunari Domen 2019a and 2019b). Solar energy is an inexhaustible clean energy source that can significantly decrease the overall energy utilization required for water splitting (Haiqing et al. 2018).

**Fig. 7:**
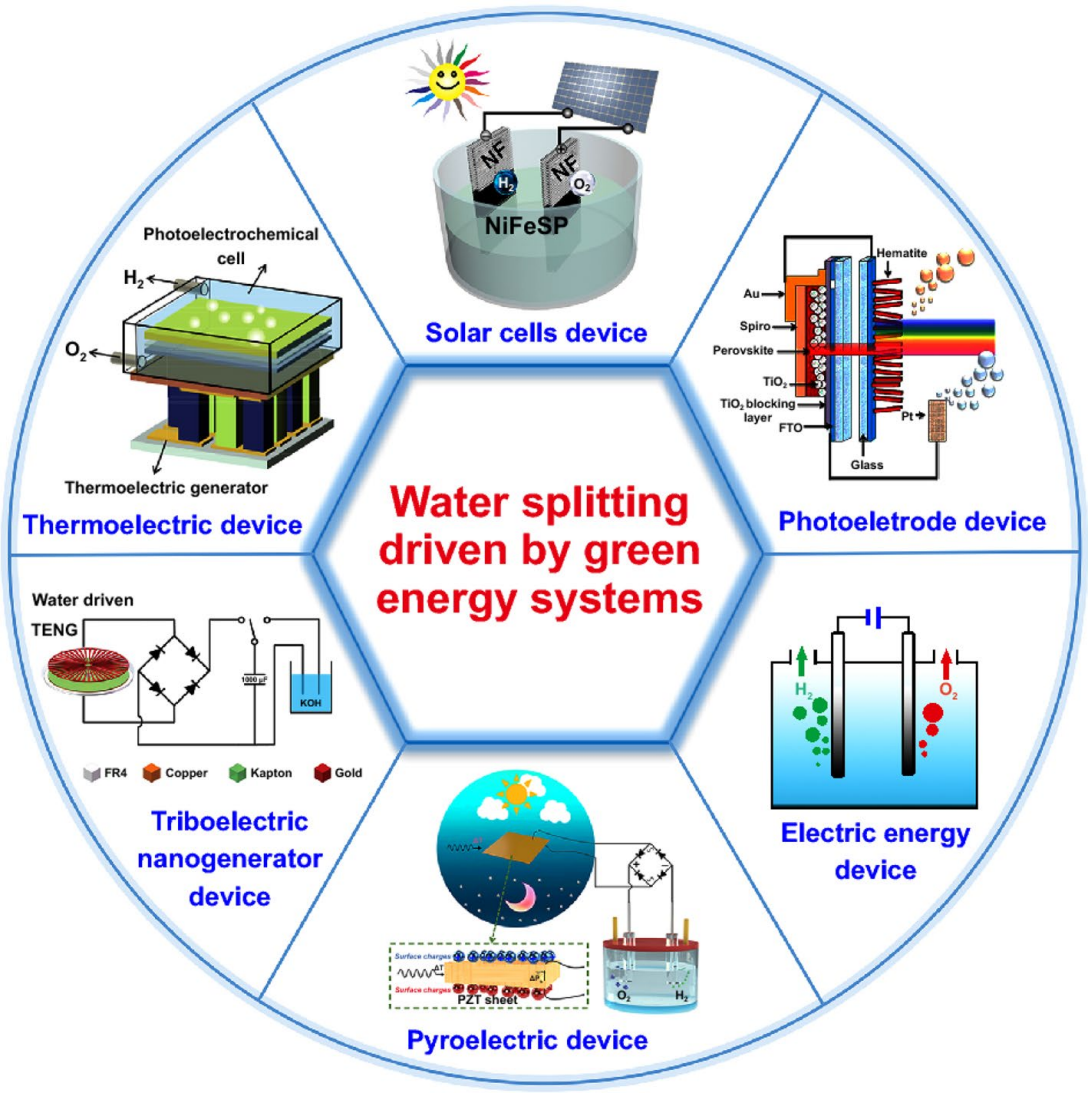
Water splitting driven by different green energy systems (Li et al., 2020)

For instance, external energy consumption can be minimized using solar cells that directly absorb the sunlight and yield voltage as an alternative to external power supply. Moreover, sunlight can be used as a heating source to generate the power needed in the water-splitting process using thermoelectrical (TE) devices (Haiqing et al. 2018). On the other hand, the natural vast availability of tidal and wind energy that can be harvested by triboelectric nanogenerators (TENGs) to generate electricity that in turn can efficiently decrease the need for external energy demand. Thus, hydrogen production via the water-splitting process can be driven using solar cells, photoelectrode, TE, TENG, along with other devices such as pyroelectric, as illustrated in Fig. 7 (Li et al., 2020).

### Electrocatalysts for water splitting

Ni, Cu and Fe are the common elements recovered from the electronic board and have shown excellent electrocatalytic activity in several electrochemical reactions in the application of energy conversions. Particularly, water electrochemical splitting reactions outperformed all other approaches upon using the appropriate catalyst (Karthik et al., 2022). Among the non-noble metal catalysts, Ni and Mo revealed a hydrogen evolution activity that is on par with the activities of the hydrogen evolution of noble metal catalysts. Those metals are also widely utilized in the electronics manufacturing industry and have been classified by the IUPAC as endangered (Karthik et al., 2022).

**Fig. 8:**
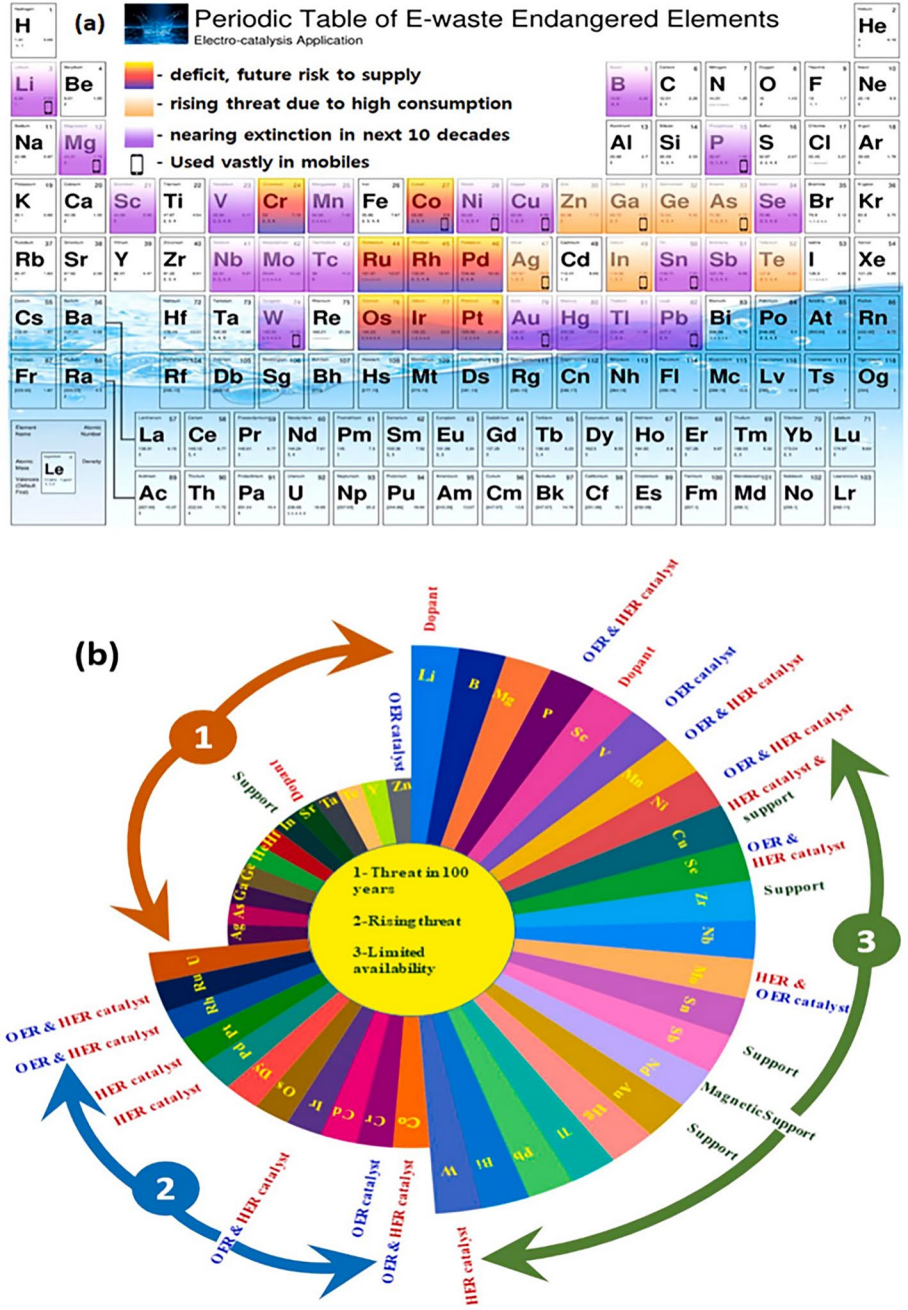
**a** IUPAC enlarged periodic table of elements (Karthik et al., 2022) and **b** the corresponding Aster plot showing the plausible water splitting properties. Reproduced with permission from Elsevier

Hence, in order to obtain an industrial profit-oriented water-splitting process, they need catalysts that can be recycled from electronic waste. Recently, a study reported the recycling of the e-waste to obtain a catalyst for HER with an appreciable overpotential at 10 mA cm^−2^ of 178 mV for H and overpotential for OER of 220 mV with stability up to 36 h (Jothi et al., 2018). According to the recent IUPAC conference, the elements are classified by researchers according to their usage and availability (Karthik et al., 2022). They unveiled elements extensively used as raw materials in the fabrication of electronic devices. For instant, In, Ag, Zn, Ga, Te, As, and Ge are expected to extend in the coming 100 years and are classified as a serious threat. Co and Cr are experiencing a rising threat due to extensive use, and Cu, Mg, Ni, Li, and Mn are classified as future supply risks due to limited availability. These declared endangered elements possess outstanding electrocatalytic water-splitting activities, as indicated in Fig. 8a and b (Karthik et al., 2022).

### Metals extracted from e-waste as catalysts for hydrogen production

Yi et al*.* (Jothi et al., 2018) reported the utilization of Cu recovered from waste electric cables as a practical and cost-effective potential current collector for overall water splitting reaction. They reached superior electrochemical performance toward both HER and OER with high replicability and stability using a facile electrodeposition process, where an efficient nickel–cobalt phosphide (NiCoP) electrocatalyst that was formed on the scrap Cu wire (SCW). Therefore, the integration of the non-noble catalyst on the SCW enhanced both the OER and the HER activities in alkaline media at10 mA cm^−2^, reaching 220 mV and 178 mV, respectively, exceeding the activity of the state-of-the-art electrocatalyst for OER and HER (IrO_2_/RuO_2_ and Pt, respectively). Additionally, an alkaline electrolyzer was stimulated by monitoring the electrocatalytic activity of the prepared electrodes at a current density of 10 mA cm^−2^ and a low voltage of 1.59 V for one day.

Slabon et al*.* (Lee et al., 2012) prepared a mixed graphene support cobalt aluminum borate (AlBCoO_3_^2+^) electrocatalyst from waste Li-ion battery cathodes. The specimens were supported on carbon (named as sample 1), and the other was supported on copper tape (named as 1C). The 1C sample was directly green prepared using the treated decomposed battery with HCl, then neutralizing the recovered materials with the extract with Na_2_CO_3_. Finally, the reaction was reduced in the presence of graphene using sodium borohydride. The prepared catalyst achieved a hydrogen production rate of 49.3 L (H_2_) min^−1^ g^−1^ Co at 70 °C with a minimum turnover number of 1.2 × 10^7^ molecules of hydrogen per Co atom. The other catalyst prepared without the C support (Sample 1) exhibited a low turnover number and showed poor electrocatalyst activity. The STEM and SEM analysis of both prepared samples concluded that the presence of carbon enhances the mass activity. Table 5 surveys the electrocatalysts derived from e-waste used in hydrogen production (Karthik et al., 2022).

**Table 5:** A survey of recovered electrocatalysts from e-waste recovered for water splitting. Permission from Elsevier

Source	Electrocatalyst		Endangered elements	References
	Activity	Stability		
Waste Cu cable wires	OER (*η* = 390 mV @ 100 mA/cm^2^ and 275 mV @ 20 mA/cm^2^)	_	Cu	(Jothi et al., 2018)
Spent Li-ion Battery (LiCoPO_4_ & LiCoO_2_)	OER (*η* = 330 mV)	C.A. @ 2.5 and 1.7 V Ag^− 1^ for 2 h	Co and Li, P	(Lee et al., 2012)
Dry cell battery (MnO_2_)	OER (*η* = 525 mV @ mA/cm^2^)	^−^	Mn	(Karthik et al.)
PCB board and wire (Cu and Ni)	HER (*η* = 178 mV) and OER (*η* = 220 mV) @ 210 mA/cm^2^	C.A. @ 1.46 V and 15 mA/cm for 36 h	Ni, Cu	(Jothi et al., 2018)
PCB	HER current density as high as 2.6 mA/ cm^2^ at—0.6 V_Ag/AgCl_		Cu	(Nekouei et al., 2021)
Waste multilayer ceramic capacitors containing BaTiO_3_, Ag, Pd, Ni and Sn	HER (156.7 μmolg^−1^ h^−1^)	–	Ag, Pd, Ni, and Sn	(Niu & Xu, 2019)
Waste tantalum capacitors	HER (200.8 μmolg^−1^ h^−1^)	–	Ta	(Niu et al., 2020)
Waste ceramic capacitor	Z-scheme enhanced H_2_ evolution rate, which was 22.2 times higher than that of g–C_3_N_4_	–	Ag, Pd, Ni, and Sn	(Babar et al., 2019)
MEMS (AlScN)	OER (*η* = 40 mV)	C.P @ 10 mA/cm for 2 h with *η* = 430 mV	Sc	(Wygant et al., 2016)
Memory device (VO_x_)	VO_x_/NiS/NF; OER ( *η* = 330 mV @ 50 mA/cm^2^)	*E* = 1.2–1.7 V	V	(Gonçalves & Araki, 2020)
Power electronics (GaN)	HER (9.4 × 10^4^ s^−1^)	CP electrolysis, stable for 8 h	Ga	(Ni et al. 2019)
Photocell, rectifier and xerography (Se –Te, Se on SS)	OER (*η* = 270 mV @ 10 mA/cm^2^)	C.A. @ 10 mA/cm^2^ for 3 h and *η* = 290 mV	Se	(Qian et al., 2019)
Dielectric in gates and laser device (Y_2_O_3_)	OER (*η* = 190 mV)	C.A. @ 1 mA/cm^2^ for 8 h & 1.5 V	Y	(Kim et al., 2017)
FET (MoS_2_)	HER (*η* = 110–254 mV @ 10 mA/cm^2^)	E = + 0.2 to − 0.3 V	Mo	(Benck et al., 2014)
Ag contact in solar cells	HER (k_o_ = 6 × 10^–12^ cm s^−1^)		Ag	(Campbell et al., 2009)
Ni–Cd battery	OER (*η* = 266 mV @ 10 mA/cm^2^)	C.P for 100 h @ 20 mA/cm^2^ and η = 300 mV	Ni and Cd	(Chen et al., 2020)
IC, chips, and processors (Sb)	HER (starts at 90 mV)	Electrolysis at − 1.1 vs Fc/Fc^+^ for 2 h	Sb	(Jiang et al., 2017)

### The use of recovered materials from e-waste for energy storage

The enduring improvement of nanomaterials to obtain more advanced nanotechnology requires a continuous raw materials supply (Klaine et al., 2012). Consequently, the nanomaterial recovery from any application of nanotechnology is essential to achieve sustainable waste management that maintains the supply of the continuous raw material demand. Energy storage applications such as supercapacitors and rechargeable batteries rely on nanomaterials. The used materials in the super capacitive application are expensive. For instant, the cost of cobalt oxide increased from 20 $/Kg in 1998 to 60 $/Kg in 2017 (Freitas & Garcia, 2007). In electrical storage devices, metal oxides are used as they have a high capacitance value which makes them good candidates. Ruthenium oxide was the first material studied due to its high surface area and conductivity (Aboelazm et al., 2021), showing an excellent capacitance of 1340 F g^−1^ that is very close to its optimum theoretical capacitance of 1400 F g^−1^ (Hu et al., 2004).

Various transition metal sulfides, oxides, hydroxides, and their respective composites have been investigated as supercapacitor materials, such as CuO, Co_3_O_4_, V_2_O_5_, MnO_2_, MoS_2_, Fe_3_O_4_, and NiO (Atef et al., 2018; Nathan et al., 2008). Co_3_O_4_ and MnO_2_ showed significant theoretical capacitance among all the investigated transition metal oxides (TMOs) exhibiting capacitances of 3560 F g^−1^ and 1360 F g^−1^, respectively, besides they have multivalence states (Gomaa et al., 2018). The ultra-layered Co_3_O_4_ catalyst has a capacitance of 548 F g^−1^ (Sumanta et al. 2011). Subsequently, recovering cost-effective nanomaterials as catalysts from e-waste management has made waste recycling an indispensable necessity. MnO_2_ is a recognized excellent pseudocapacitive nanomaterial that can substitute expensive and toxic candidates, particularly RuO_2_. Despite the Mn high abundance in nature, it is crucial to prevent abusing the natural resources to develop the technologies. On the contrary, the best way to obtain MnO_2_ is through the Mn recovery from waste materials.

United States Environmental Protection Agency (USEPA) reported that about 160,000 tons of batteries are generated, and the annual Mn recovery could reach up to 20,000 tons (María et al. 2013). Moreover, a great amount of Mn in waste batteries can be used as a supercapacitor electrode (Sayilgan et al., 2009). Gam et al. reported the use of MnO_2_ nanoflowers recovered from Zn–C Batteries (Gomaa et al. 2014). The recovered MnO_2_ electrocatalyst exhibited a great capacitance of 294 F g^−1^, exhibited high performance in 1.0 M Na_2_SO_4_ electrolyte and showed high stability up to cycles of 88% (Gomaa et al. 2014). The same group used cyclic voltammetry conversion as an alternative electrochemical technique for porous MnO_2_ nanoflowers recovery using spent Zn–C batteries. The process includes thermal treatment of 900 °C powder by cyclic voltammetry for 1000 cycles at 50 mV s^−1^ in the potential window—0.1—0.9 V. They stated that the capacitance reached 309 F g^−1^ with high stability of 93% was reported (Gomaa et al. 2017).

Co_3_O_4_ has been extensively examined in the application of the electrode design of supercapacitors since it has multiple oxidation states and high conductivity. Aboelazm et al. managed to recover Co_3_O_4_ nanomaterials with high active surface area from discarded Li-ion batteries using a magnetic field. The recovered cobalt oxide showed a superior capacitance of 1273 F g^−1^ with 96% cycling stability (Eslam et al. 2018). Furthermore, Rajkumar et al. reported the recovery of copper oxide nanoparticles from spent circuit boards though the leaching of the powdered circuit boards with a mixture of 37% HCl and 63% HNO_3,_ followed by the addition while stirring of NaOH. The precipitate was then filtered, purified and dried for 6 h at 80 °C. The performance of the recovered Cu–O supercapacitor was elucidated. The fabricated electrocatalyst electrode exhibited a capacitance of 408 F. g^−1^ at 1 Ag^−1^ and retaining a capacitance that is 93.1% of its initial value after three thousand GCD cycles (Rajkumar et al., 2022) (Table 6).

**Table 6:** A survey of recovered electrocatalysts from e-waste recovered for supercapacitor

Waste	Recovered Material	Preparation Method	Morphology	Specific capacitance (F g^−1^)	Stability (%) @ cycles	References
Zn–C Batteries	MnO_2_	Leaching and electrowinning	Nano-flower	208.5 @ 0.1 A g^−1^ 294 @ 10 mV s^−1^	88 @ 900	(Gomaa et al. 2014)
	MnO_2_	Cyclic voltammetry conversion	Nano-flower	09 @ 0.1 A g^−1^	93 @ 1650	(Gomaa et al. 2017)
	MnO_2_	thermal treatment	Spherical & cubic shape	125 Fg^−1^ at 5 mVs^−1^	80% @ 2100	(Farzana et al., 2019)
	MnO_2_/rGO	Cyclic voltammetry conversion	Nano-flower/nanosheets	473 Fg^−1^ @ 0.25 A g^−1^	95 @ 2000	(Gomaa et al., 2018)
Spent alkaline battery cathode	MnO_2_	Electrodeposition	spherical cluster shape	549 Fg^−1^ @5 mV s^−1^	87.2 @ 5000	(Thomas et al. 2019)
LIB	Co_3_O_4_	Chemical precipitation and heat-treatment	Agglomerated crystals	13 Fg^−1^ @ 1 mV s^−1^		(E.M.S. Barbieri, et al., 2014)
	Co_3_O_4_	Magnetic electrodeposition	Well-defined hierarchical Nanostructure	1273 Fg^−1^ @ 1 A g^−1^	96 @ 5000	(Eslam et al. 2018)
	Reduced graphene oxide (rGO)	Simple chemical method	intrinsic flake-like morphology	112 Fg^−1^ @ 0.5 Ag^−1^	108@ 20,000	(Subramaniaet al. 2018)
	LiCoO_2_	Chemical precipitation	Crystal structure	654 F g ^−1^	86.9% @ 4000	(Xu et al., 2015)
	MnCo_2_O_4_	Chemical precipitation		122.6 F g^–1^ @ 5 mV s ^–1^	94.4% @8000	(Natarajan et al., 2022)
	Li–Ni–Mn–Co hydroxide	Electrodeposition	Nanoflowers-like structure	951 F g^–1^ @ 1 Ag	90% @ 10,000	(Mesbah et al., 2020)
Printed circuit boards	CuO	Simple chemical method		408 F g^− 1^ @ 1 Ag^− 1^	93.1% @ 3000	(Rajkumar et al., 2022)
SIM card	CuO	Simple chemical method	Cauliflower-like structure	542 Fg^−1^ @ 1 Ag^−1^	95.3% @5000	(Rajkumar et al., 2020)
Waste Toner	Derived Carbon/Fe_3_O_4_	thermal treatment	Sphere in shape	536 Fg^−1^ @ 3 Ag ^−1^	97% @5000	(Kaipannan et al., 2019)

### E-waste recycling for sustainable development goals

During the last two decades, sustainability has become a principal part of a principal global agenda. Key events such as Brundtland Commission’s report, Kyoto Protocol, the Millennium Development Goals in 2000, and recently the Paris Climate Treaty in 2015 showed the significance of sustainable practices. Consequently, businesses have considered serious action inside and outside their companies regarding sustainability issues hence augmenting attention in associating the business responses to sustainability. Sustainability initiatives by companies are on the rise, as an evidence by recent reports. Companies have recently tried to identify numerous ways to move toward a sustainable management system, such as cost-reduction profits, waste minimization, and improved resource management to sustain the raw materials for the coming generation (Bhaskar, 2018).

**Fig. 9:**
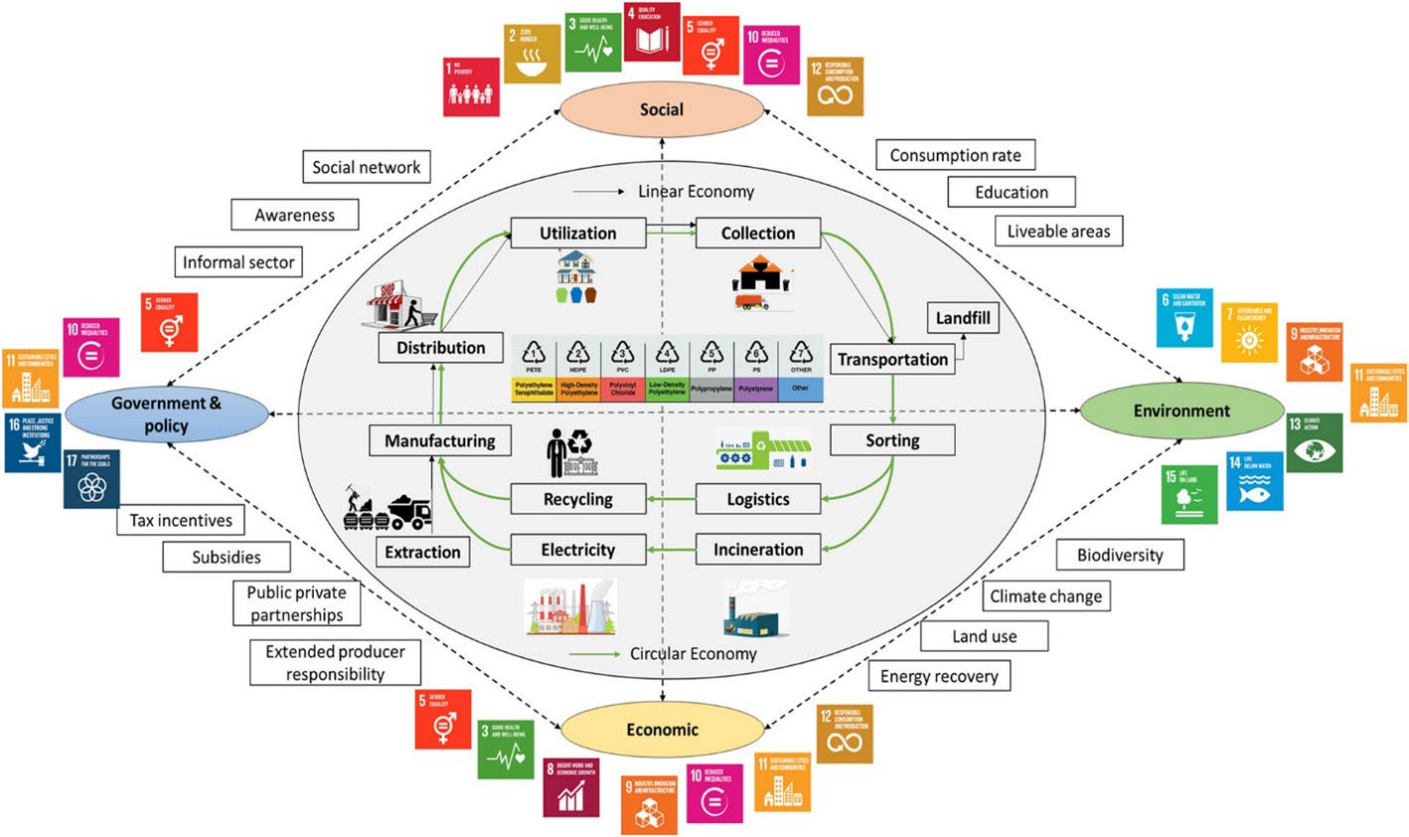
Circular economy approaches in the management of electronic waste (Sharma et al., 2021) with permission from Elsevier

In 2015, the United Nations members embraced the sustainable development agenda for 2030 agenda which contains 17 goals with 169 targets for achieving this plan for sustainable development strategy (Kumar et al., 2017b). Firms worldwide are directed to align their business to meet a minimum of one of the recent sustainable design goals. The definition of sustainability is broad and challenging for establishments to comprehend and implement (Dao et al., 2011). The accumulative consumption rates of electronic devices, low lifetime, little availability of repairing options, and high remote working consequences increase demands for vigorous practical infrastructure and digital renovation across the firm's models (Rautela et al., 2021). Electronic waste is anticipated to experience a sudden increase due to the recent COVID-19 pandemic (Rautela et al., 2021). The irregular disposal methods of electronic waste lead to several health and environmental hazards since it contains hazardous and toxic elements (Rautela et al., 2021). From an economic point of view, E-waste is considered an ‘urban mine’ as it includes several precious metals, such as Fe, Au, and Cu, that can be recovered and reused as secondary raw materials, as illustrated in Fig. 9 (Sharma et al., 2021).

Globally, recycled e-waste was approximately $57 billion in 2019 (Forti et al., 2020). Thus, capturing the remaining cost or utility of the e-waste is essential to reach a circular economy in the electric and electronics production industry. Therefore, recycling e-waste materials significantly increases the sustainability of primary raw materials and reduces greenhouse gas emissions (Sustainable Development Goals 13). The total equivalent amount of CO_2_ emitted only from discarded air conditioners and fridges to the atmosphere is 98 Mt, which was not handled according to an environmentally sound manner (Sustainable Development Goals 3, 11, 12, 13). This amount of CO_2_ emissions is almost 0.3% of global emissions reported in 2019 (Forti et al., 2020).

Electronic waste management thoroughly narrates various sustainable development goals. For instants, Goal 3: Health and well-being. This goal covers illness and death rates associated with hazardous materials exposure from air, soil and water. Goal 6: Sanitation and clean water. This goal pursues attaining nontoxic clean drinking water as well as the removal of hazardous materials. Goal 8: Economic growth and decent work that targets entrepreneurship and innovation. Goal 11: Sustainable cities and communities emphasizing per-capita environmental impact. Goal 12: Responsible production and consumption that focuses on the life cycle assessment of each product and its impact on the environment, besides the waste prevention and reduction techniques for individual products (Sharma et al., 2021).

In contrast, a more precise sub-indicator must be performed to monitor the growth of the electronic waste such as firms’ models through which the producer preserves the responsibility of their products, enhancing the recovery rate (Morseletto, 2020). Adapting these models could increase the amount of captured materials used in electronic applications while emerging a new relationship with the customers and increasing sustainability for the coming generations.

Global energy consumption increases as a result of the worldwide increase in the population. Consequently, the energy demand increased from 5.52 × 10^20^ to 6.07 × 10^20^ J in 2010–2015 and is expected to be 6.97 × 10^20^ J by 2030 (IRENA, 2018). The current population relies on supplying the energy demand depending on fossil fuels, resulting in drastic climate problems (Acheampong et al., 2016). Consequently, it was a must to inspire clean energy to substitute fossil fuels. It was planned to supply 20% of the global energy demand from clean renewable energy resources in 2014 (Acheampong et al., 2016). Investment in clean renewable energy sources such as solar, wind, hydro and thermal energy has been considered essential to obtaining affordable electricity by 2030 (Barasa et al., 2018). To attain this goal, a reduction in the global domestic and industrial consumption of electricity is required to be 14% and adapting a cost-effective technology. The developing countries are encouraged to surge their infrastructure and develop energy conversion and storage technologies to offer clean energy, which can encourage political growth, and socioeconomic and environmental improvement. Besides, renewable energy can increase employment opportunities to reach 24 M by 2030 (IRENA, 2018; Sen & Ganguly, 2016).

The global sustainability goals have three key pillars: renewable energy sources, increasing energy access, and enhancing energy efficiency, as well as addressing the five following targets (Acheampong et al., 2016; Bishoge et al., 2018): 1. Equality in the accessibility of reliable, cheap, and new energy facilities. 2. Augmented stake of global renewable energy compared to non-renewable energy. 3. Doubling the global population rate while maintaining appropriate energy efficiency. 4. Elevation of international collaboration to license investment in different renewable energy technologies. 5. Green energy technologies improvement to enrich the supply of feasible and modern energy facilities to all communities.

## Conclusion and outlook

The world market for electrical and electronic equipment (EEE) has grown exponentially in recent years while the lifespan of these products has been decreasing. The end-of-life of these products has been in landfills and recycling centers, creating new challenges among stakeholders. Government entities and politicians are attempting to provide solutions for the hazardous components disposed off in landfills. Furthermore, scientists and international organizations are working to develop various processes for recycling valuable materials for use in renewable energy technologies. Electronic devices contain many valuable materials and, when appropriately extracted, can be more valuable than those present in the ore. Policymakers have begun implementing e-waste management policies that allow technology transfer to recover high-quality materials from different types of e-waste. This would help them to meet the sustainable development and Paris Agreement goals. This review described the various hydrometallurgical methods used to extract materials from printed circuit boards and lithium-ion batteries.

Structural changes in the world’s energy system, primarily relying on renewable energy, can start with the important role of renewable energy in combination with energy efficiency. Technology development beyond research creates an enabling environment that includes education and awareness-raising. The appropriate and reliable mix of the materials extracted from electronic waste as an eco-design catalyst in different renewable energy technologies has emerged as a hot topic. As explained in the review, some researchers started by recovering copper from printed circuit boards and its use for supercapacitors and water-splitting techniques. However, further research is needed to recover other materials such as Cobalt, Iron, Manganese, and Lithium and use them in various renewable energy technologies. From the presented analysis, future directions may include:

The use of precious metals, copper, and other materials extracted from electronic waste as catalysts for CO_2_ reduction, ammonia production, and green hydrogen production.

The use of the extracted materials in renewable energy technologies, such as green biogas production, due to its high methane production efficiency.

The use of recovered materials from e-waste for water treatment and desalination.

## Data Availability

The datasets used and analyzed during the current study are available from the corresponding author upon reasonable request.
